# Evolution of antiviral resistance captures a transient interdomain functional interaction between chikungunya virus envelope glycoproteins

**DOI:** 10.1128/mbio.02530-25

**Published:** 2025-10-29

**Authors:** Leandro Battini, Sara A. Thannickal, Malena Tejerina Cibello, Mariela Bollini, Kenneth A. Stapleford, Diego E. Álvarez

**Affiliations:** 1 Instituto de Investigaciones Biotecnológicas, Universidad Nacional de San Martín (UNSAM)–Consejo Nacional de Investigaciones Científicas y Técnicas (CONICET) 28221 , San Martín, Argentina; 2 Laboratorio de Química Medicinal, Centro de Investigaciones en Bionanociencias (CIBON), Consejo Nacional de Investigaciones Científicas y Técnicas (CONICET) 62873, Buenos Aires, Argentina; 3 Department of Microbiology, New York University Grossman School of Medicine 12296, New York, New York, USA; 4 Escuela de Bio y Nanotecnologías (EByN), Universidad Nacional de San Martín, San Martín, Argentina; Okayama University, Kurashiki, Okayama, Japan

**Keywords:** alphavirus, antivirals, viral envelope glycoproteins, membrane fusion

## Abstract

Envelope proteins drive virus and host-cell membrane fusion to achieve virus entry. Fusogenic proteins are classified into structural classes that function with remarkable mechanistic similarities. Membrane fusion implies coordinated movements of protein domains through a series of sequential steps. Structures for the initial and final conformations are available for several fusogens, but folding intermediates remain largely unresolved, and the interdependency between regions that drive conformational rearrangements is not well understood. Chikungunya virus (CHIKV) particles display heterodimers of envelope proteins E1 and E2 associated as trimeric spikes that respond to acidic pH to trigger fusion. We followed the experimental evolution of CHIKV under the selective pressure of a novel entry inhibitor. Mutations arising from selection mapped to two residues located in the distal domains of the E2 and E1 heterodimer and spikes. Here, we demonstrate that the antiviral mechanism involves inhibition of membrane fusion. Phenotypic characterization of recombinant viruses indicated that the selected mutations confer a fitness advantage under antiviral pressure, and that the double-mutant virus overcame antiviral inhibition of fusion while single mutants were sensitive. In addition, molecular dynamics simulations suggest that these two residues modulate the conformational rearrangement of the E1-E2 heterodimer. In this line and supporting a functional link between residues, the double-mutant virus displayed a higher pH threshold for fusion than single-mutant viruses. Finally, mutations resulted in distinct replication and spreading outcomes in mice and infection rates in mosquitoes, underscoring the fine-tuning of envelope proteins’ function as a determinant for the establishment of infection. Altogether, our approach captured an otherwise unresolved interdomain interaction.

**IMPORTANCE:**

Chikungunya virus (CHIKV) is a reemergent pathogen that has caused large outbreaks in the last 20 years. There are no available antiviral therapies, and a vaccine has only recently been approved. We describe the mode of action of an inhibitor designed to target CHIKV envelope proteins, blocking entry at the stage of fusion between the virus envelope and host membranes. Fusion is common to the entry of enveloped viruses. Virus envelope proteins drive fusion, undergoing a series of transitions from an initial metastable conformational state to a more stable post-fusion state. Intermediate conformations are transient and have mostly remained inaccessible to structure determination. Here, a selection of viruses that are resistant to antiviral inhibition of fusion uncovered a functional interaction between two residues residing in domains that are apart in both the pre-fusion and post-fusion states. Thus, we provide new insight into the molecular detail of the inner working of virus fusion machinery.

## INTRODUCTION

Chikungunya virus (CHIKV) is transmitted to humans by mosquitoes. Infection commonly results in acute fever and joint pain that can progress into chronic polyarthritis (1, 2). Since 2004, CHIKV has spread throughout the tropical and subtropical regions around the world, causing outbreaks associated with high socioeconomic cost (3, 4). Genomic adaptation of the virus to new mosquito vectors has been pointed out as one of the major causes for the geographical spread of the virus (1, 5, 6). Therefore, the study of viral determinants of CHIKV fitness in its natural hosts may provide valuable information regarding the continued adaptation of the virus.

CHIKV is an alphavirus of the *Togaviridae* family (7). It has a 12 kb positive-stranded RNA genome that encodes for 10 proteins in two open reading frames (ORFs). The second ORF in the 3′ of the genome encodes for the structural proteins of the virus: the capsid (C) and the envelope glycoproteins (E3-E2-E1). E2 and E1 are transmembrane proteins that together with E3 form a heterotrimer on the surface of the viral particle, mediating the interaction with cell receptors and fusion between viral and endosomal membranes during entry, respectively (8). E2 belongs to the immunoglobulin superfamily of proteins and folds into three globular domains (A, B, and C) connected by two antiparallel β-strands, referred to as β-ribbon (9). E1 is a class II viral fusion protein and folds into a β-sheet-rich structure with three β-barrel domains (I, II, and III) bearing the fusion loop in the tip of domain II (9, 10). In the viral particle, three E3-E2-E1 trimers fold into a viral spike, with E1 located next to the membrane and E2 positioned over E1, protecting the fusion loop (11).

CHIKV enters the cell by receptor-dependent endocytosis. In the final stage of the entry process, the decrease in endocytic pH induces membrane fusion, triggering a major conformational rearrangement of the envelope proteins that involves dissociation of E1 and E2 and reassociation of E1 into homotrimers (9, 12–15). The joint action of multiple E1 homotrimers is required to generate the force necessary to curve the opposing membranes (10, 16). For this reason, the conformational rearrangement in different spikes must be coordinated, and the timing of each step must be tightly regulated.

We have previously identified compound 11 as a small molecule inhibitor of CHIKV infection following a virtual screening against the envelope proteins of the virus (17). We showed that compound 11 inhibited the internalization of the viral particle and selected a viral variant resistant to the antiviral activity of the compound, which harbors mutations in E1 (Y24H) and E2 (P173S). Interestingly, whereas recombinant viruses carrying both E1-Y24H and E2-P173S showed the resistant phenotype, neither of the single mutants did, suggesting a functional interaction between these two residues located in distant regions of CHIKV envelope glycoproteins.

In this study, we show that compound 11 inhibits the pH-dependent membrane fusion process. Based on molecular dynamics simulations of the pre-fusion conformation of the envelope proteins, we propose that residues E1-Y24 and E2-P173 are associated with kinetic barriers that act as checkpoints of the protein conformational change during the fusion process. In this line, characterization of recombinant viruses showed that combined E1-Y24H and E2-P173S mutations altered pH dependence for fusion compared to both WT and single E1-Y24H and E2-P173S mutant viruses. In turn, experimental infection resulted in enhanced replication in mice infected with the double mutant virus but impaired ability to infect mosquitoes compared to the WT virus. Altogether, the results presented in this work support a functional interaction between residues E1-Y24 and E2-P173 that function in a concerted manner to regulate the fusion process and impact on the establishment of infection *in vivo*.

## RESULTS

### Compound 11 inhibits membrane fusion

**Fig 1:**
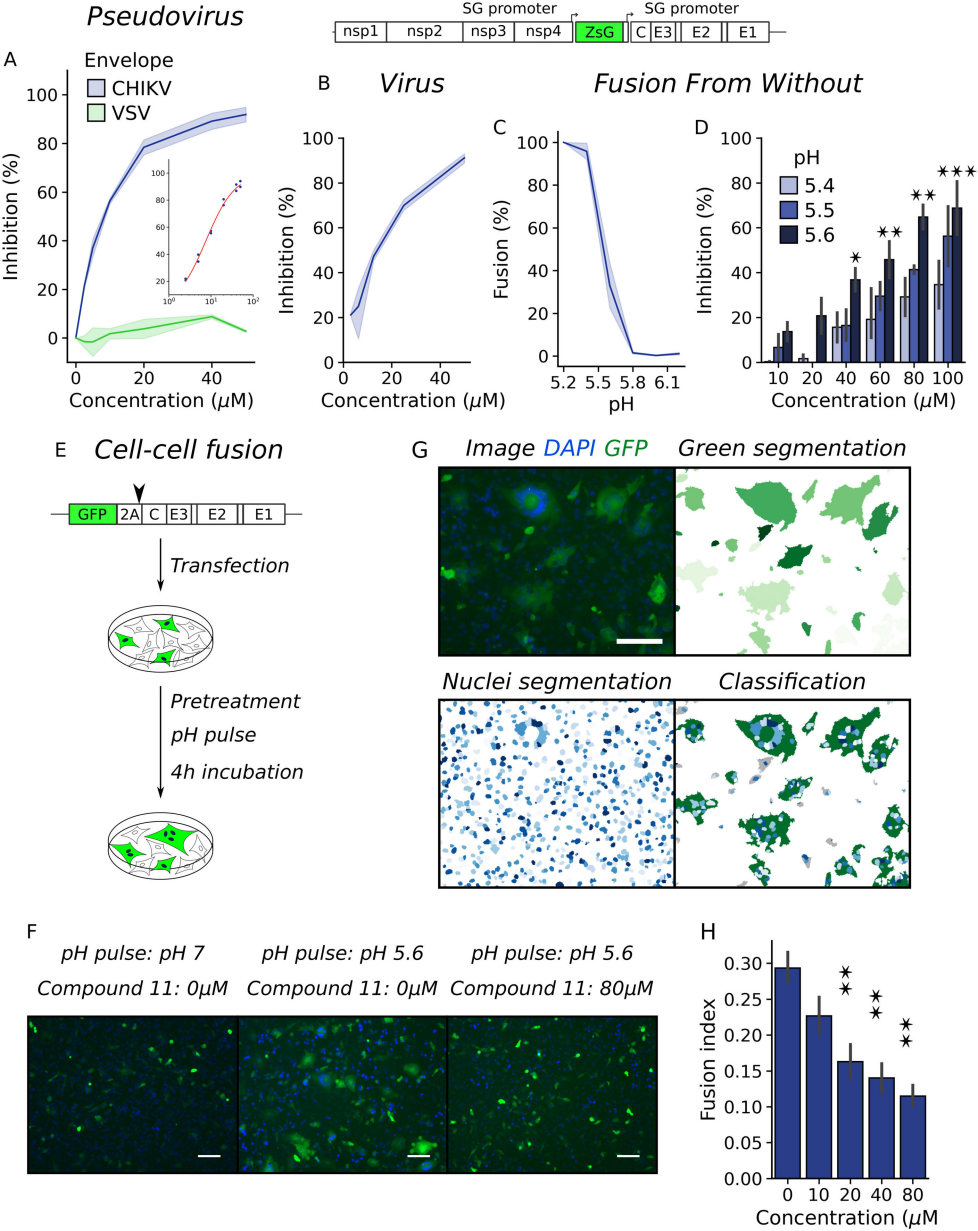
Compound 11 inhibits the fusion process during CHIKV entry. (**A**) Inhibition of pseudotyped lentivirus by compound 11 in BHK cells. Results represent the mean and standard error of two independent experiments. (**B**) Schematic representation of CHIKV ZsGreen genome (top). Dose response curve for the inhibition of infectious foci formation of WT CHIKV ZsGreen in BHK cells treated with increasing concentrations of compound 11 (bottom). Data points represent the mean and standard error of three independent experiments. (**C**) FFWO assay with WT CHIKV ZsGreen virus in BHK cells. Fusion was triggered with citric acid in phosphate-buffered saline (PBS) titrated at different pHs. Results represent the mean and standard error of the percentage of infected cells as determined by flow cytometry relative to the percentage at pH 5.2 of three independent experiments. (**D**) FFWO assay with CHIKV WT in the presence of compound 11. Results represent the mean and standard error of the inhibition of infected cells relative to the untreated control of four independent experiments. One-way ANOVA with Tukey’s *post hoc* test. Asterisks indicate significant differences against the untreated control at each pH. (**E**) Schematic representation of pCI-neo-GFP-CHIKV C-E2-E1 construct and workflow of cell-cell fusion assay. (**F**) Representative images obtained after different treatments in the cell-cell fusion assay. The white scale bar represents 100 µm. (**G**) Image analysis of cell-cell fusion assay. From the original image, green cells and nuclei were segmented, and green cells were classified into non-fused cells and syncytium. The fusion index was calculated as fusion index = 1 − number of green cells/number of nuclei. (**H**) Fusion index in the cell-cell fusion assay against increasing concentration of compound 11. Results represent the mean and standard error of three independent experiments. One-way ANOVA with Tukey’s *post hoc* test. *P < 0.5, **P < 0.01, ***P < 0.001.

We have identified a small molecule inhibitor of CHIKV infection designed to target a pocket located behind the fusion loop of E1 in a cleft formed between domain II of E1 and domains A and B of E2. Time of drug addition assays showed that compound 11 inhibited the production of viral progeny when added at the time of infection and that the effect was gradually lost when addition was delayed, indicating that treatment blocked virus entry but not the release of infectious particles. In line with this observation, we found that the compound had no effect on virus attachment and specifically inhibited internalization, reducing relative infection by threefold in Vero cells treated with 50 µM of compound 11 (17). Moreover, the approach suggested that treatment halted viruses at a post-attachment stage and inhibited further progress of infection as the virus was not able to propagate from treated cells to non-treated cells in a focus-forming assay. To further characterize its mode of action, we tested the inhibitory activity of the compound against lentiviruses pseudotyped with CHIKV or VSV envelope proteins using BHK cells as a target for transduction (Fig. 1A). Virus pseudotypes bear CHIKV or VSV envelope proteins surrounding an HIV capsid and allow testing for the specific inhibition of envelope protein function by compound 11 at the stage of entry since no further rounds of virus replication occur after primary infection. While the VSV pseudotype was resistant to the antiviral activity of compound 11, it inhibited CHIKV pseudotyped lentiviruses (EC_50_ = 7.6 ± 0.1 µM, Fig. 1A inset) with an EC_50_ similar to that obtained against a reporter CHIKV expressing ZsGreen from a subgenomic promoter (EC_50_ = 13.77 ± 1.87 µM and Fig. 1B), demonstrating that the compound specifically inhibited CHIKV entry into the host cell. Moreover, both CHIKV and VSV are endocytosed through the clathrin-dependent pathway (18, 19). As we have shown that compound 11 did not interfere with virus attachment (17), the fact that inhibition was specific against the CHIKV pseudotype rules out an indirect effect of the compound in endocytosis and suggests that compound 11 may inhibit the function of CHIKV envelope proteins in the membrane fusion process. Then, we performed fusion from without (FFWO) assays in which the fusion of viruses attached on the cell surface is triggered directly at the plasma membrane by lowering the pH of the medium. As a result, the degree of infection is dependent on the fusogenic activity of the viral particles. We first set up the assay by triggering fusion at different pHs after the absorption of CHIKV-ZsGreen (Fig. 1C). Data showed that the fusogenic activity of the envelope proteins followed a sigmoidal curve with a fusion threshold around pH 5.5, similar to the behavior described in literature (20, 21). We then evaluated the effect of adding compound 11 at increasing concentrations in FFWO triggered at pH 5.4, 5.5, or 5.6. We found that compound 11 inhibited fusion in a concentration-dependent manner and had a stronger effect at higher pH (Fig. 1D).

As a complementary approach, we set up a cell-cell fusion assay based on the ability of recombinant CHIKV envelope proteins exposed on the plasma membrane to induce fusion with target cells. In BHK cells transfected with a reporter construct encoding for GFP and CHIKV structural proteins separated by a self-cleaving peptide, green multinuclei syncytia formed after treatment with low pH medium (Fig. 1E and F). Fluorescence image analysis was followed to classify GFP-expressing cells based on the number of encompassed nuclei, and the fusogenic activity was estimated using a fusion index (fusion index = 1 − cells/nuclei) that ranges from 1 to 0, with higher values indicating a higher fusogenic activity (Fig. 1G). As in the FFWO assay, compound 11 inhibited the fusogenic activity of CHIKV envelope proteins in a concentration-dependent manner when fusion was triggered at pH 5.6 (Fig. 1H).

In conclusion, complementary approaches using virus pseudotypes, fully infectious reporter CHIKV, and protein expression constructs to evaluate the function of CHIKV envelope proteins demonstrate that compound 11 inhibited entry of CHIKV into the host cell and pinpoint the mechanism of action to the inhibition of the fusion process driven by CHIKV envelope proteins.

### E2-P173S E1-Y24H double mutant resistant phenotype is associated with overcoming of fusion inhibition

**Fig 2:**
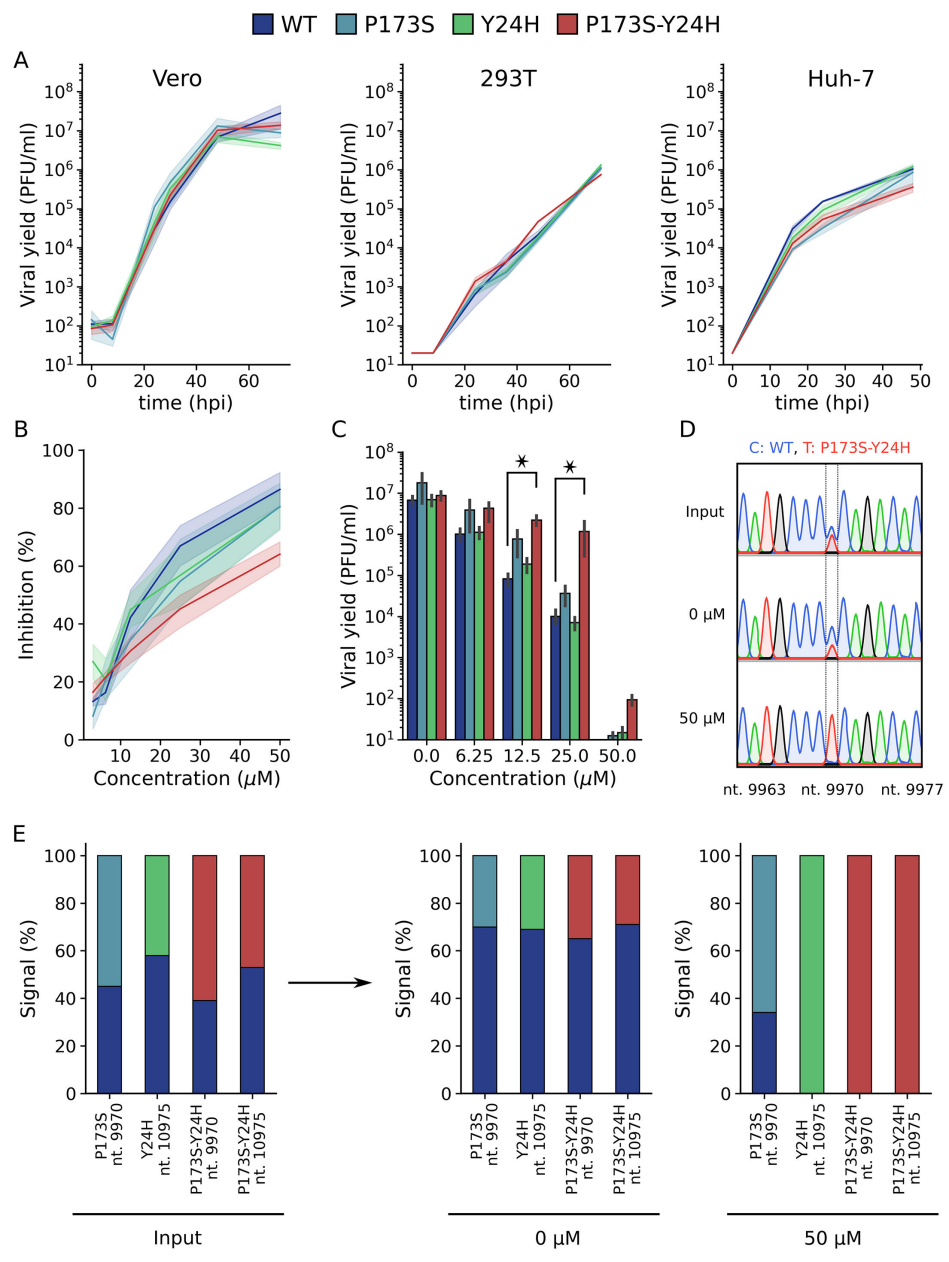
Single-mutant E1-Y24H and E2-P173S, and double-mutant viruses outcompete wild-type virus growth in the presence of compound 11. (**A**) Growth curve of WT and mutant viruses in Vero, HEK 293T, and Huh-7 cells in the absence of compound. Results represent the mean and standard errors of at least two independent experiments. (**B**) Dose response curves for the inhibition of infectious foci formation of WT and mutant CHIKV ZsGreen in Vero cells treated with increasing concentrations of compound 11. Data points represent the mean and standard error of three independent experiments. (**C**) Viral yields were measured 48 hours after the infection of Vero cells with WT and mutant viruses and after the treatment with increasing concentrations of compound 11. Results represent the mean and standard error of three independent experiments. Kruskal-Wallis with Dunn’s *post hoc* test. *P < 0.05. (**D**) Representative chromatograms obtained in the competition assay. The nucleotide position is indicated in the *x*-axis label. WT and mutant viruses were mixed at an initial 1:1 ratio and used to infect Vero cells that were not treated (0 µM) or treated at 50 µM of compound 11. The supernatants were collected at 72 hours after infection. Following RNA extraction and RT-PCR, the composition of the resulting virus population was assessed by Sanger sequencing. The peak height was used to determine the ratio between viruses. (**E**) Quantification of the competition assay. Signal percentages represent the ratio of the peak height for a given nucleotide divided by the sum of the heights of the nucleotides in the mixed signal. The nucleotide position is indicated in the x-axis label.

Evolution of antiviral resistance generally occurs through stepwise selection of mutations that often result in reduced viral fitness. To study the impact of E2-P173S and E1-Y24H double and single mutants on the resistant phenotype, we used recombinant viruses that were previously constructed in the reporter CHIKV-ZsGreen background (17). We first assessed virus replication kinetics in Vero cells. Growth curves showed that all viruses behaved in the same manner as WT (Fig. 2A, left). Similarly, mutations did not alter growth kinetics in human 293T or Huh-7 cells (Fig. 2A, middle and right). To assess fitness in the presence of compound 11, we measured antiviral activity using reporter CHIKV-ZsGreen in a focus-forming assay. WT and single mutants E2-P173S and E1-Y24H showed similar sensitivity to compound 11, displaying EC_50_ of 11.6 ± 3.4 µM, 15.7 ± 1.2 µM, and 11.2 ± 2.1 µM, respectively, while the double mutant displayed EC_50_ = 21.4 ± 1.3 µM (Fig. 2B), indicating that only the combined mutations confer a partially resistant phenotype to compound 11 antiviral activity. The phenotype was further confirmed when viral yields were measured following infection of cells treated with increasing compound concentrations (Fig. 2C). As expected, increasing compound concentrations resulted in a gradual decrease in virus yields. The trend for single mutants was similar to WT, showing a ~1,000-fold decrease in viral titers in cells treated at 25 µM of compound compared to non-treated cells. The threefold difference in titer of the E2-P173S mutant compared to WT in the absence of compound was also observed across the concentrations tested, suggesting that the two viruses are similarly sensitive to antiviral treatment. In contrast, the E2-P173S E1-Y24H double-mutant virus demonstrated a clear resistance to the antiviral activity of compound 11, displaying more than 100-fold higher viral yields at 25 µM of compound compared to WT. Taken together, focus forming and virus yield assays indicate that development of antiviral resistance was associated with selection of the combined E2-P173S and E1-Y24H mutations.

To further understand the contribution of selected mutations to antiviral resistance, we evaluated the fitness of WT and mutant viruses in virus competition assays. Untreated cells or cells treated with 50 µM of compound 11 were infected with WT and mutant viruses mixed at an initial 1:1 ratio. Then, the composition of the resulting virus population was assessed by Sanger sequencing of the RT-PCR product amplified from cell culture supernatants by comparing the peak height of the WT and mutant alleles (Fig. 2D). As expected, we observed a 1:1 signal ratio for input populations. In untreated cells, although mutant alleles were detected, in all cases, the ratio favored the WT, suggesting that mutations impaired virus fitness. In turn, in treated cells, both E1-Y24H and the double-mutant viruses completely displaced the WT virus, and the signal ratio for the E2-P173S and WT was inverted compared to competition in untreated cells, indicating that the mutant allele conferred an increment in fitness in the presence of compound 11 (Fig. 2E). Focus-forming and virus yield assays did not anticipate the association of individual mutations to resistance, which became evident in direct competition assays under stringent antiviral pressure. In turn, the assay also suggested that mutations have a cost in virus fitness when viruses compete without antiviral pressure, suggesting that these conflicting forces would have acted together in the selection process that led to the emergence of the double-mutant virus at high antiviral concentrations.

**Fig 3:**
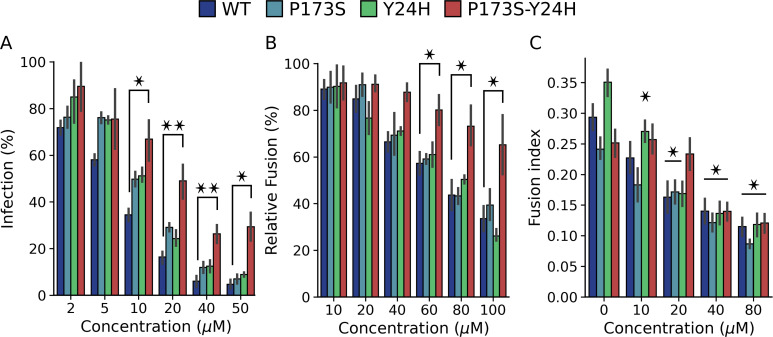
E1-Y24H E2-P173S double-mutant escapes compound 11 fusion inhibition. (**A**) Inhibition of WT and mutant pseudotyped lentivirus with increasing concentrations of compound 11. Results represent the mean and standard error of the percentage of infected cells relative to the untreated control of three independent experiments. One-way ANOVA with Tukey’s *post hoc* test. (**B**) Fusion inhibition of compound 11 in the FFWO assay (pH 5.6) with WT and mutant viruses. Results represent the mean and standard error of the percentage of infected cells relative to the untreated control of three independent experiments. One-way ANOVA with Tukey’s *post hoc* test. (**C**) Fusion inhibition of compound 11 in the cell-cell fusion assay (pH 5.6). Results represent the mean and standard error of the fusion index of three independent experiments. One-way ANOVA with Tukey’s *post hoc*. Differences between each concentration and the untreated control for each virus. *P < 0.05, **P < 0.01.

Next, we sought to gain insight into the mechanism by which the double-mutant virus overcomes the antiviral activity of compound 11. First, we evaluated the effect of compound 11 on infection with WT, and single- and double-mutant pseudotyped lentiviruses to directly test the impact of mutations on virus entry (Fig. 3A). At concentrations greater than 10 µM, only the double-mutant virus displayed increased levels of infection compared to WT, indicating that the combined E2 P173S E1-Y24H mutations in the envelope proteins are sufficient to confer antiviral resistance.

Next, we performed FFWO and cell-cell fusion assays with the WT and single- and double-mutant viruses to determine whether the mutations allow the virus to overcome fusion inhibition by compound 11. In the FFWO assay, the single mutants behaved in the same manner as the WT virus, showing a dose-dependent inhibition of infected cells (Fig. 3B). In contrast, the double mutant infected a higher percentage of cells in the range of concentrations tested. In line with these results, in the cell-cell fusion assay, the fusogenic activity of the WT and both single mutants decreased as drug concentrations increased (Fig. 3C). Contrarily, there were no differences in the fusogenic activity of the double mutant up to 20 µM of compound. Compared to the FFWO assay, resistance of the double mutant virus to the inhibitory effect in the cell-cell fusion assay was not observed at the highest concentrations tested, likely due to methodological differences between approaches based on infection with replicative viruses and overexpression of envelope proteins, respectively.

Altogether, these results show that resistance of E2-P173S E1-Y24H double mutant to the antiviral activity of compound 11 is associated with a weaker inhibition of the fusogenic activity of CHIKV envelope proteins, reinforcing the notion of a functional interaction between E2-P173 and E1-Y24.

### E2-P173S and E1-Y24H are located near two important hinges of CHIKV envelope glycoproteins

**Fig 4:**
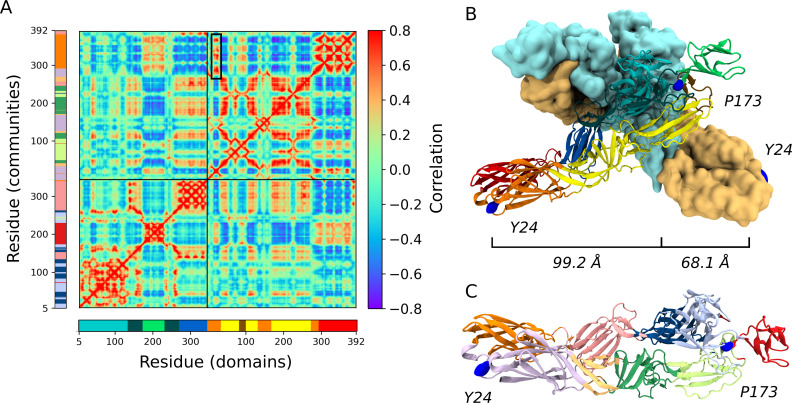
E2-E1 pre-fusion heterodimer structure and dynamics. (**A**) DCCM obtained from the molecular dynamics trajectory. The DCCM represents the correlation of motion between each pair of residues in the simulation. The black box shows the correlated motion between the E1-Y24 loop and domain E1-III. (**B**) Mapping of E2-P173 and E1-Y24 on the trimeric spike. Representation of the spike formed by three E2-E1 heterodimers (PDB: 6NK7). One heterodimer is represented as a cartoon, and the remaining two are shown as surfaces. Domain E2-A is colored in cyan, E2-B in green, E2-C in blue, E1-I in orange, E1-II in yellow, and E1-III in red. The β-ribbon is colored in dark cyan and the fusion loop in ochre. E2-P173 and E1-Y24 are represented as blue surfaces. (**C**) Envelope proteins colored by communities, detected by the Girvan-Newman algorithm, form the RIN.

It is noteworthy that E2-P173S and E1-Y24H are apart from each other both in the E2-E1 heterodimer (99.2 Å) and in the viral trimeric spike (68.1 Å). To gain deeper insights into the role of E2-P173 and E1-Y24 and the functional interaction between them, we performed molecular dynamics (MD) simulations of the envelope proteins in the pre-fusion conformation (22), and constructed and analyzed the residue interaction network (RIN). To build the RIN, we computed the contact matrix and the dynamic cross correlation matrix (DCCM) of the Cα atoms from the MD trajectory (Fig. 4A) (23). We used RIN to calculate residue communities using the Girvan-Newman algorithm (24) and mapped the resulting communities in the envelope proteins’ crystal structure. Communities represent groups of residues that move in a concerted manner. Detected communities were associated with the envelope protein domains but were not identical (Fig. 4B and C).

**Fig 5:**
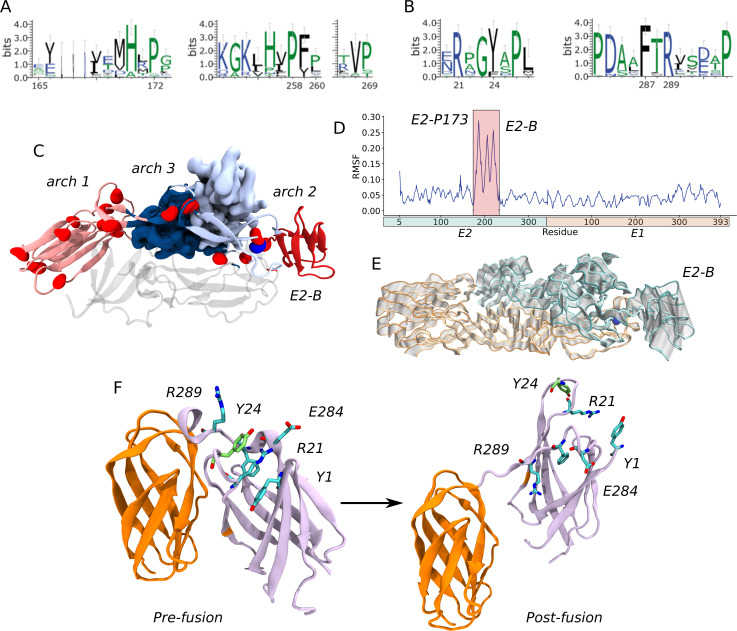
E2-P173 and E1-Y24 are located near flexible hinges in the envelope proteins of CHIKV. (**A** and **B**). Amino acid alignment of alphavirus E1 and E2 envelope proteins. Alignments were generated using MAFFT (48) and the following sequences: Semliki Forest complex (SFV): chikungunya virus IOL (CHIK IOL; JF274082.1), O'nyong-nyong virus (ONNV; NC_001512.1), Semliki forest virus (SFV; NC_003215.1), Ross River virus (RRV; GQ433354.1), Bebaru virus (BEBV; HM147985.1), una virus (UNAV; HM147992.1), Mayaro virus (MAYV; NC_003417.1); aquatic virus complex (Aquatic): southern elephant seal virus (SESV; NC_016960.1), salmon pancreas disease virus (SPDV; NC_003930.1), sleeping disease virus (SDV; NC_003433.1); eastern equine encephalitis complex (EEEV): eastern equine encephalitis virus (EEEV; NC_003899.1), Madariaga virus (MADV; KJ469622.1); Venezuelan equine encephalitis complex (VEEV): Venezuelan equine encephalitis virus (VEEV; NC_001449.1), Everglades virus (EVEV; NC_038671.1), pixuna virus (PIXV; NC_038673.1), Tonate virus (TONV; NC_038675.1), Cabassou virus (CABV; NC_038670.1), Rio Negro virus (RNV; NC_038674.1), Mosso das Pedras virus (MDPV; NC_038857.1); mosquito-specific virus complex (Mosquito): Trocara virus (TROV; NC_043402.1), Eilat virus (EILV; NC_018615.1); western equine encephalitis complex (WEEV): Sindbis virus (SINV; NC_001547.1), Whataroa virus (WHATV; NC_016961.1), western equine encephalomyelitis virus (WEEV; NC_003908.1), Aura virus (AURV; NC_003900.1). (**A**) Residue conservation in the complementary strands of the β-ribbon next to arches 2, 3, and 1. (**B**) Residue conservation in the loop of domain I (left) and the flexible linker between domains I and III (right). Sequence logos were generated with Weblogo 3 (49). (**C**) E2-P173 is located in a hinge region in the β-ribbon. E2 domains C, B, and the β-ribbon are represented as cartoons, E2-A is represented as a surface, and the tip of E1 is represented as transparent cartoons. Prolines on E2 are represented as red spheres (E2-P173 blue). The protein is colored according to MD communities. (**D**) RMSF per residue across the second principal component (PC) of a PCA of the MD trajectory. (**E**) Movement across the second PC. The protein is represented as tubes, and the extreme conformations are colored in orange for E1 and cyan for E2. The conformations in between are represented as transparent tubes. (**F**) The E1-Y24 loop interacts with the flexible linker between domains E1-I and E1-III. Only domains E1-I and E1-III are represented for the pre-fusion (PDB 3N42) and post-fusion (PDB 1RER) conformations and colored according to MD communities. The residues involved in the interaction network with E1-Y24 in the pre-fusion conformation are represented as sticks and are separated from each other in the post-fusion conformation.

Next, we analyzed the position of each residue in the protein and in the RIN. E2-P173 is located between E2-B and the β-ribbon. This proline is conserved in viruses of the Semliki fever virus complex and adjacent to E2-P172, which is conserved in all alphaviruses (Fig. 5A). The PP motif is associated with a break in secondary structure. Additionally, in the context of the E1-E2 heterodimer, these residues are located next to arch 2 in the complementary strand of the β-ribbon (Fig. 5C). The association between prolines and arches in the complementary strands of the β-ribbon is also present in arch 1 (E2-P269) and arch 3 (E2-P258 and E2-P260). Interestingly, these regions correspond to breakpoints between residue communities and, thus, represent breakpoints in residue connectivity (Fig. 4A and 5C). In fact, E2-P173 is located at the hinge of the second Principal Component (PC) of the envelope proteins in a PCA of the MD trajectory, which is the PC associated with the highest RMSF of E2 domain B (Fig. 5D and E). Overall, these data suggest that P172 and P173 would control the flexibility of the β-ribbon and E2 domain B, which is essential in the first steps of the conformational rearrangements of the envelope glycoproteins. Thus, E2-P173S may alter the behavior of the WT protein in this regard.

In turn, in the MD simulations, there was a strong interaction between the loop bearing E1-Y24 and the flexible linker between domains I and III (residues 283–294). Both loops were part of the same community (Fig. 5F, pre-fusion) and showed a high correlation between each other and with domain E1-III (Fig. 4A black box, Table 1). E1-Y24 is an aromatic residue and establishes π-cation interactions with two nearby arginines (E1-R289 and E1-R21). Interestingly, alphaviruses display an aromatic residue at position 24 (tyrosine or phenylalanine) and an arginine is strictly conserved at positions 289 and 21, indicating that this network of interactions is common across the genus (Fig. 5B). In turn, E1-R21 interacts with E1-E284, E1-F287, and E1-Y1, building an interaction network that may be responsible for the correlated motion of the loop and the flexible linker. Based on residue conservation, alphaviruses would maintain the pattern of π-cation interactions between an aromatic residue in the E1-24 position and positively charged residues at positions E1-289 and E1-21. E1-Y24 loop is separated from E1-III and the flexible linker in the post-fusion conformation of E1 envelope protein (Fig. 5F, post-fusion). This means that the interaction between the E1-Y24 loop and the linker has to break during the conformational change that drives the fusion process. Thus, the stability of this interaction may be important for the stability of the pre-fusion conformation of E1 and the regulation of the fusion process. Histidine residues become protonated at the acidic pH of the endosome; thus, the E1-Y24H substitution may impact the strength of the interaction network connecting this residue to the linker in the context of the pH-triggered fusion process.

**TABLE 1:** E1-Y24 interaction network^*a*^

Residue i	Residue j	E_coul_^*b*^	E_LJ_^*c*^	E_total_^*d*^	Correlation^*e*^
Y24	R21	−0.4 ± 0.6	−16.7 ± 1.3	−17.1 ± 1.9	0.906
	P22	−2.2 ± 0.2	−6.1 ± 0.2	−8.3 ± 0.4	0.913
	P26	−0.1 ± 0.1	−3.4 ± 0.1	−3.5 ± 0.1	0.914
	E284	−10.8 ± 3.4	−5.5 ± 0.6	−16.2 ± 4.0	0.656
	F287	0.5 ± 0.2	−10.2 ± 0.2	−9.7 ± 0.4	0.838
	T288	−11.5 ± 0.1	−6.7 ± 0.1	−18.2 ± 0.2	0.873
	R289	1.7 ± 0.2	−11.4 ± 0.2	−9.7 ± 0.4	0.938
	V290	−1.0 ± 0.1	−6.9 ± 0.1	−7.9 ± 0.1	0.942
R21	Y1	0.3 ± 0.2	−14.0 ± 1.6	−13.7 ± 1.8	0.553
	G23	−1.9 ± 0.1	−3.2 ± 0.1	−5.1 ± 0.2	0.919
	Y24	−0.4 ± 0.6	−16.7 ± 1.5	−17.1 ± 2.1	0.906
	S26	0.1 ± 0.1	−0.9 ± 0.1	−0.7 ± 0.1	0.842
	E284	−70.7 ± 6.8	−0.2 ± 1.0	−70.9 ± 7.8	0.558
	F287	−1.4 ± 0.3	−10.5 ± 0.5	−11.9 ± 0.8	0.69

^*a*^ Interaction energy and correlation of motion between Y24 and R21 with other E1 residues in the molecular dynamics simulations.

^*b*^ Coulomb interaction energy.

^*c*^ Lenard-Jones interaction energy.

^*d*^ Total interaction energy.

^*e*^ Correlation of motion between residues.

Altogether, MD simulations suggest that while E2-P173 would be associated with the flexibility of domain B, E1-Y24 may modulate the stability of E1 prefusion conformation. In this manner, both residues could be associated with important kinetic barriers in the conformational rearrangement of the envelope proteins and, thus, would play a role in the regulation of the timing and coordination of the different steps in the fusion process. If this were the case, substitutions associated with antiviral resistance may alter the stability and fusogenic function of the E1-E2 heterodimer and would have arisen together as compensatory mutations. Altogether, our analysis provides a mechanistic hypothesis that could explain the molecular basis behind the functional interaction of E1-Y24 and E2-P173 observed in antiviral resistance.

### Impact of E2-P173S and E1-Y24H on viral particle stability and envelope proteins functionality

**Fig 6:**
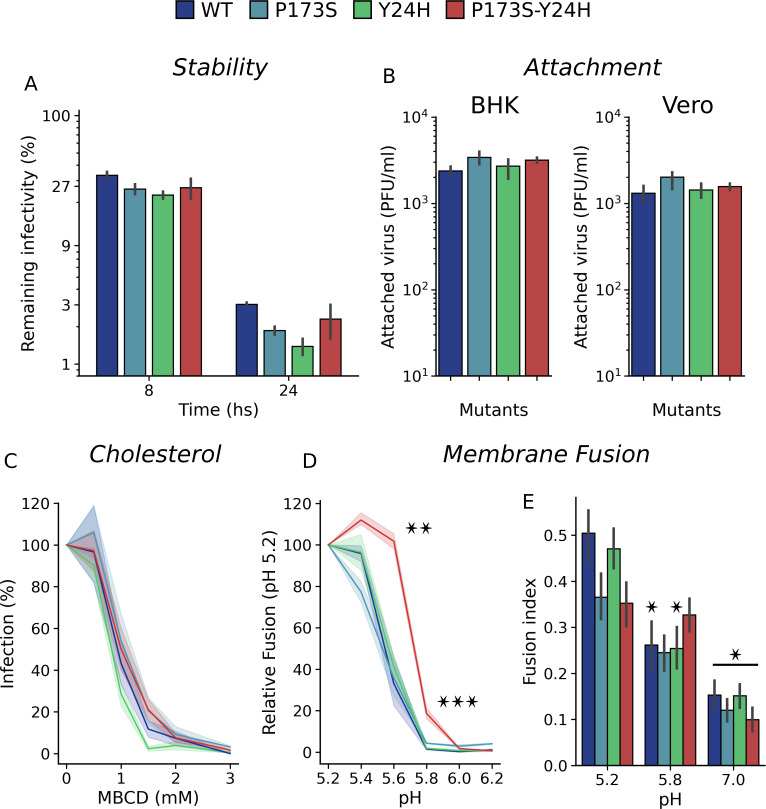
The impact of E1-Y24H and E2-P173S on the envelope protein is associated with a shift in fusion pH threshold. (**A**) Thermostability of WT and mutant viruses. Viruses were incubated at 37°C in cell culture medium, and the remaining infectivity after 8 or 24 hours was quantified using plaque assays. The figure displays virus titers at 8 or 24 hours compared to initial titers as percentages. (**B**) Attachment to BHK and Vero cells. BHK and Vero cells were incubated with WT and mutant viruses for 1 h at room temperature, and the attached virus was quantified by plaque assay after cell lysis. Results represent the mean and standard error of the number of attached viruses from three independent experiments. (**C**) Cholesterol dependence. BHK cells were treated with increasing concentrations of MβCD for 1 hour at 37°C and infected with WT or mutant CHIKV-ZsGreen viruses. Following infection, 20 mM NH_4_Cl was added to prevent further rounds of virus entry, and the percentages of infected cells were measured 1 day post-infection by flow cytometry. Results represent the mean and standard error of the percentage of infected cells relative to the untreated control from three independent experiments. (**D**) FFWO assay. Membrane fusion was triggered by the treatment with phosphate-buffered saline (PBS) citric acid solution titrated at different pH. Results represent the mean and standard error of the percentage of infected cells relative to pH 5.2 for each virus from three independent experiments. One-way ANOVA with Tukey’s *post hoc* test. Differences between each mutant and the WT for each pH. (**E**) Cell-cell fusion assay. Results represent the mean and standard error of the Fusion Index of two independent experiments. One-way ANOVA with Tukey’s *post hoc* test. Differences against pH 5.2 for each mutant. *P < 0.05, **P < 0.01, ***P < 0.001

To test our working hypothesis linking resistance-associated mutations to the coordinated regulation of the envelope protein complex dynamics, we next studied the thermal stability of the viral particle and the functionality of the envelope proteins of WT and mutant viruses. To study thermal stability, we quantified infectivity after incubation of viruses at 37°C in cell culture medium (Fig. 6A). All viruses showed a similar stability profile, with approximately 25% of the initial infectivity retained after 8 hours of incubation and 2% after 24 hours, indicating that mutations have no major impact on stability.

Initial characterization of virus growth showed no differences in virus yields between WT and mutant viruses (Fig. 2A), suggesting that mutations do not alter CHIKV assembly or release. To further characterize the impact of mutations on protein functionality, we studied their effect on viral attachment, cholesterol dependence for CHIKV entry, and fusion. Virus attachment assays showed no differences between mutant and WT viruses in either BHK or Vero cells (Fig. 6B).

Target membrane cholesterol was shown to promote endocytic uptake of CHIKV, and mutations in E1 were previously linked to an increased cholesterol dependency for fusion (5). To measure the cholesterol dependence for viral infection, we used methyl-β-cyclodextrin (MβCD) to capture cholesterol and lower its levels in the plasma membrane (25) (Fig. 6C). MβCD was thoroughly washed prior to infection to address cholesterol-dependent entry and minimize the impact of cholesterol depletion on nsp1 plasma membrane anchoring and subsequent RNA replication (26, 27). All viruses were similarly sensitive to MβCD, suggesting that the WT and mutant viruses display a similar cholesterol dependence.

Finally, we assessed the fusogenic activity of WT and mutant envelope proteins. In the FFWO assay (Fig. 6D), the fusion degree against pH followed a sigmoidal curve with a marked fusion threshold for WT and mutant viruses. Both single mutants showed a fusion profile similar to the WT virus. In contrast, there was a clear shift toward a higher pH in the threshold for fusion of E2-P173S E1-Y24H double-mutant virus. We observed a similar behavior in the cell-cell fusion assay. While the fusion index at pH 5.2 was higher for the WT and E1-Y24H mutant, at pH 5.8, the fusion index was higher for the double mutant, indicating a shift of the fusion threshold toward neutral pH for this virus (Fig. 6E).

Altogether, these results show that E1-Y24H and E2-P173S mutations qualitatively change the fusion phenotype, shifting the pH threshold for the double mutant, which confirms the functional interaction between these two residues.

### Mouse and mosquito infections with E2-P173S and E1-Y24H single and double mutants

**Fig 7:**
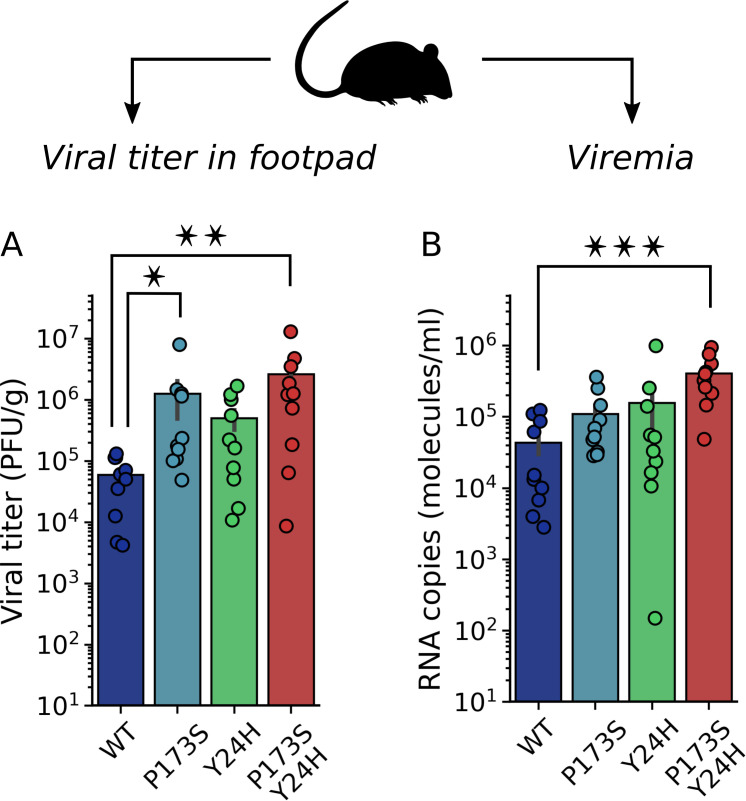
Infectivity of E1-Y24H and E2-P173S single and double mutants in mice. Four- to five-week-old male and female C57BL/6J mice (*n* = 10 per experimental group) were infected with 1,000 PFU of WT or mutant CHIKV ZsGreen via subcutaneous inoculation in the footpad. Two days post-infection, (**A**) the viral titer was determined in the footpad by plaque assay, and (**B**) the viral load was quantified in the serum by qRT-PCR. For panels A and B, Kruskal-Wallis with Dunn’s *post hoc* test, *P < 0.05, **P < 0.01, ***P < 0.001.

Previous studies indicated that altered fusion phenotypes impact virus infectivity *in vivo* (20, 21, 28, 29). Given that mutations associated with antiviral resistance changed the fusion phenotype, we decided to characterize the impact of mutations *in vivo*. We performed infections in mice with the WT and mutant viruses. Two days post-infection, we quantified footpad viral titers that reflect virus replication at the site of infection and viremia as a proxy of dissemination (Fig. 7A and B). Footpad viral titers were higher for mutant viruses than for the WT, with the double mutant displaying the highest viral yield. In this line, viremia was also higher for mutant viruses, showing a positive correlation with the infection level in the primary infection site.

**Fig 8:**
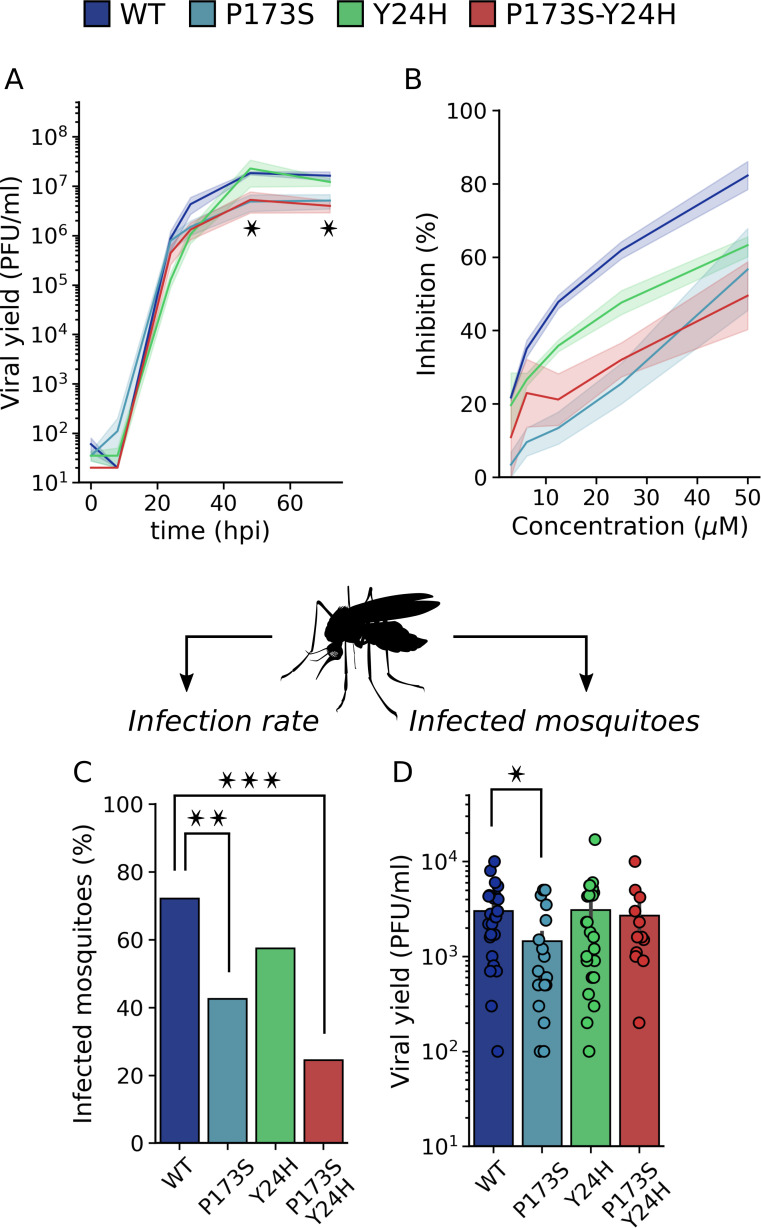
Infectivity of E1-Y24H and E2-P173S single and double mutants in mosquitoes. (**A**) Growth curve of WT and mutant viruses in C6/36 cells. Results represent the mean and standard error of three independent experiments. Kruskal-Wallis with Dunn’s *post hoc* test, *P < 0.05. (**B**) Dose response curves for the inhibition of infectious foci formation of WT and mutant CHIKV ZsGreen in C6/36 cells treated with increasing concentrations of compound 11. Data points represent the mean and standard error of three independent experiments. (**C and D**) 4- to 7-day-old female *Ae. aegypti* mosquitoes were infected with a blood meal containing 10^6^ PFU/mL of WT or mutant CHIKV-ZsGreen. Seven days post-infection, the viral titer was determined in mosquito bodies. (**C**) Percentage of infected mosquitoes. Fisher’s exact test, **P < 0.01, ***P < 0.001. (**D**) Viral yield in infected mosquitoes. Kruskal-Wallis with Dunn’s *post hoc* test, *P < 0.05.

Next, we evaluated the fitness of mutant viruses in the vector host. Similar to mammalian cell lines, CHIKV entry into mosquito cells is pH dependent. However, differences in receptor usage and alternative entry pathways have been described for the different host cell lines (30). We first carried out growth curves of WT and mutant viruses in the mosquito C6/36 (*Ae. albopictus*) cell line (Fig. 8A). We found that both E2-P173S and the double mutant reached a lower viral titer than WT (fourfold), and the growth of E1-Y24H was delayed. Profiling of the antiviral activity of compound 11 showed that the WT virus was sensitive to compound 11 (EC_50_ = 12.87 µM). As expected, the double mutant virus displayed a resistant phenotype (EC_50_ = 48.47 µM). Interestingly, single mutants were partially resistant to treatment with the E2-P173S virus displaying a higher EC_50_ (E2-P173S EC_50_ = 43.39 µM vs. E1-Y24H EC_50_ = 23.71 µM) (Fig. 8B). Finally, we assessed virus fitness following feeding of *Ae. aegypti* mosquitoes with an infectious blood meal. In line with *in vitro* data, all mutant viruses infected a lower percentage of mosquitoes than WT (Fig. 8C), with a statistically significant reduction for E2-P173S and the double mutant. Interestingly, viral titers in the bodies of infected mosquitoes were similar for the double mutant and WT (Fig. 8D), suggesting a defect for the mutant virus to overcome the midgut infection barrier to establish an infection (31). In contrast, the viral titer for E2-P173S single mutant was twofold lower than WT, suggesting an impact of the mutation on viral fitness in mosquitoes. Moreover, the result further shows an effect of E1-Y24H on the double-mutant phenotype.

Overall, these results show an opposite impact of E2-P173S and E1-Y24H on viral fitness in the alternate hosts, with a beneficial effect in mice and a detrimental effect in mosquitoes. Noteworthy, while competition experiments indicated that mutations appeared to be associated with loss of fitness in untreated cells *in vitro* (Fig. 2E), they resulted in increased fitness in mice with the double mutant displaying a more pronounced phenotype than single mutants. In turn, mutations compromised virus fitness *in vitro* and in the mosquito vector. In addition, E1-Y24H partially rescued the deleterious effect of E2-P173S in mosquitoes, altogether reinforcing the functional interaction between these two residues.

## DISCUSSION

In this work, we studied the mechanism of action of a previously identified small molecule inhibitor of CHIKV infection and characterized the phenotype of viruses carrying mutations associated with antiviral resistance. Using complementary approaches, we demonstrated that compound 11 specifically inhibits the pH-dependent membrane fusion driven by CHIKV envelope proteins during entry. Characterization of mutant viruses indicated a functional interaction between E2-P173 and E1-Y24: viruses with the combined mutations E2-P173S and E1-Y24H displayed a distinct resistance to compound 11, likely associated with a shift in the fusion threshold toward higher pH, and showed a significantly increased replication in mice and impaired rate of infection in mosquitoes. We propose that E2-P173S and E1-Y24H cooperatively act to engage the heterodimer in its conformational rearrangement at a pH closer to neutral in comparison to WT.

### Functional interaction between E2-P173 and E1-Y24 in the regulation of the fusion process

The results presented here support that E2-P173 and E1-Y24 work together in the regulation of the fusion process despite being distantly located in the envelope proteins of CHIKV. Interestingly, a connection between nearby residues has been previously observed. Infection of mosquitoes with a virus carrying an E1-V80Q mutation, close to E2-P173S, resulted in the selection of pseudorevertants with the second-site mutation E1-N20Y, near E1-Y24H (21). In the same line, the emergence of the epistatic mutations E1-K211T and E1-V156A in the context of the Indian Ocean lineage virus carrying a valine at E1-226 was associated with increased fusion at lower pH and higher sensitivity to NH_4_Cl treatment, suggesting a functional connection between residues 156, 211, and 226 in the entry dynamics (6). Taken together with our results indicating that compensatory mutations E2-P173S and E1-Y24H modulate the fusogenic function, these results suggest a link between two distant regions of the envelope complex.

Enveloped virus entry culminates with the fusion of viral and cellular membranes and the delivery of the viral genome into the host cell cytosol. Virus envelope proteins anchored to the lipid envelope perform fusion following activation triggered by an external cue such as receptor binding or exposure to acidic pH. A common pathway for fusion involves protein conformational rearrangement from an initial prefusion state to the insertion of a fusion motif in the target membrane in an extended prehairpin state and final folding back into a post-fusion hairpin state (32). Beyond the commonalities in the fusion process, virus fusion proteins diverge in structure and organization on the virus surface. Three canonical classes of fusion proteins are recognized based on their structure. Alphavirus E1 is a class II fusion protein. Cryo-electron tomography has recently allowed us to visualize the pathway of CHIKV membrane fusion, and together with cryo-electron microscopy and crystallography, allowed us to reconstruct conformational rearrangements of the mature E1-E2 heterodimer (9, 32, 33). Still, details on the dynamics of molecular transitions at the residue level remain unresolved. Under acidic conditions, the central arch of E2 β-ribbon becomes disordered and domain B opens to expose the fusion loop at the tip of E1 (12, 22, 34). E2-P173 is located in a breakpoint in residue connectivity associated with the arch 2 of the β-ribbon, and together with structural analyses, our MD simulations indicate that this structure acts as a hinge for the movement of domain B at the beginning of the fusion process. In turn, E2 dissociates from E1, which rearranges as a homotrimer in the post-fusion state. MD results showed that the E1-Y24 loop strongly interacts with residues in the flexible linker between E1 domains I and III (E1-R289, E1-R21, E1-F287, E1-Y1, and E1-E284) in the pre-fusion conformation. Importantly, this interaction is broken during the conformational change of the envelope proteins toward the post-fusion state. Interestingly, E1-F287 and E1-R289 have been identified as a part of a different interaction network that stabilizes the post-fusion E1 trimer (35), suggesting that the conformational transition of the linker could be important in the regulation of the fusion process. Altogether, we hypothesize that E2-P173 and E1-Y24 are linked in the modulation of important kinetic barriers of the conformational change of the envelope proteins of CHIKV, acting as checkpoints of the fusion process. On the one hand, E2-P173S could alter the flexibility of the arch 2 of the β-ribbon, imposing a kinetic barrier to the opening of domain B. On the other hand, protonation of E1-H24 in E1-Y24H would lower the stability of the pre-fusion interaction network that stabilizes the linker, altering the energetic barrier that E1 needs to overcome in the transition between the pre-fusion and post-fusion states. The fact that the fusion phenotype is only altered in the double mutant suggests a multistep reaction with sequential checkpoints. As the dissociation of E1 and E2, the assembly of the post-fusion trimer, and the joint action of different trimers in the formation of the fusion pore require the coordination between different E1-E2 dimers in the viral particle (10, 16), the relative rates of the different checkpoints may be fundamental for the coordination of the heterodimers.

### Impact of E2-P173S and E1-Y24H on *in vivo* infectivity

E2-P173S and E1-Y24H had opposite effects on fitness in experimental infection of mosquitoes and mice. They resulted in impaired infection rates in mosquitoes but enhanced viral loads in mice relative to the WT virus. Further supporting a connection between these residues, in both mosquitoes and mice, the phenotype was more pronounced for the double mutant. Interestingly, the results highlight the evolutionary barrier imposed over viruses that alternate between hosts to develop resistance to an antiviral in the natural infection cycle (36). Mutations that confer resistance in the mammalian host are not necessarily adaptive in the alternate host and may even have a deleterious effect (37), preventing the fixation of the mutation in a natural population.

In further studies, it would be interesting to address the link between the change in the fusion phenotype, an increase in the fusion threshold, and the modulation of the viral fitness in the natural hosts. Notably, an opposite relationship between the fusion phenotype and the fitness in mosquitoes was observed in previous studies, where E1-V80L/Q mutants displayed a decreased fitness in mosquitoes associated with a shift of the fusion threshold toward more acidic pH (20, 21). These results suggest a complex relationship between the different functions of the fusion machinery that warrants further investigation.

In conclusion, in this study, we characterized the mechanism of action of a small molecule inhibitor of CHIKV and showed that the compound inhibits the membrane fusion process during CHIKV entry. Additionally, the study of mutations associated with resistance to the antiviral activity of compound 11 identified a functional interaction between two distant residues in the envelope proteins of the virus that, together, impact the fusion phenotype and the *in vivo* fitness of CHIKV.

## MATERIALS AND METHODS

### Cells and viruses

Vero (*Cercopithecus aethiops* kidney, ATCC CCL-81), HEK-293T cells (human embryonic kidney cells expressing SV40 T antigen, ATCC CRL-3216), and Huh-7 cells (human hepatoma cells, provided by Apath LLC) were grown in Dulbecco’s modified Eagle’s medium (DMEM; Gibco). BHK cells (*Mesocricetus auratus* hamster kidney, ATCC CCL-10) were grown in MEM alpha medium (Gibco). All mammalian cell lines were grown at 37°C in a 5% CO_2_ atmosphere in medium supplemented with 10% fetal bovine serum (FBS), 100 U/mL of penicillin, and 100 µg/mL of streptomycin (Gibco). C6/36 cells (*Aedes albopictus* larvae, ATCC CRL-1660) were grown at 28°C in Leibovitz L-15 medium (Gibco) supplemented with 10% FBS, 10% of tryptose phosphate (29.5 g/L, Britania), 100 U/mL of penicillin, 100 µg/mL of streptomycin, and 250 ng/mL of amphotericin B (Gibco).

WT and mutant CHIKV-ZsGreen of the Indian Ocean Lineage (Eastern Central and South Africa genotype) were derived from infectious cDNA clones as previously described. Viral titers of the final stocks were determined by plaque assay. Viral stocks were stored at −70°C until use. The construction of E1-Y24H and E2-P173S single and double mutants is described elsewhere (17).

### Compound

The synthesis and purification of compound 11 were previously described (17). For biological assays, compound 11 was first dissolved in dimethyl sulfoxide (DMSO) to a final concentration of 10 mM and then diluted in culture medium to the appropriate concentration. In all assays, the DMSO concentration was lower than 1%.

### Pseudotypes production and inhibition assay

The CHIKV ORF encoding for viral structural proteins (C-E3-E2-6K-E1) was cloned into pCI-Neo (Promega) to obtain pCI-neo-CHIKV.

HEK-293T cells at 70% confluence were co-transfected with plasmids psPAX2 (10 µg, Addgene #12260), pLB-GFP (10 µg, Addgene #11619), and pCI-neo-CHIKV or pMD2.G (2 µg, Addgene #12259) using polyethylenimine (PEI) reagent. Pseudotypes were harvested 2 days post-transfection and were concentrated by centrifugation at 3,000 × *g* 4°C overnight.

For the determination of compound 11 inhibitory activity of pseudotype infection, BHK cells at 50% confluence were treated with increasing concentrations of compound 11 and were infected with pseudotypes of CHIKV or VSV at a multiplicity of infection (MOI) of 0.05. 10 µg/mL of Polybrene (Sigma) was added to enhance pseudotype adsorption. Three days after infection, the percentage of infected cells was quantified by flow cytometry.

### Fusion from without

Confluent BHK cells were treated with increasing concentrations of compound 11 and infected with CHIKV-ZsGreen WT or mutant viruses at an MOI of 0.1 at room temperature. Then, the inoculum was removed, and a phosphate-buffered saline (PBS) and citric acid solution was titrated to an acidic pH, with the corresponding concentration of compound 11 added. After incubating for 2 minutes at room temperature, the acidic medium was removed, cells were washed with PBS, and α-MEM 20 mM NH_4_Cl was added. Cells were analyzed by flow cytometry at 1 day post-infection.

### Cell-cell fusion

BHK cells at 80% confluence grown on a coverslip were transfected with the pCI-neo-GFP-CHIKV C-E2-E1 plasmid using PEI reagent. One day post-transfection, cells were treated with increasing concentrations of compound 11 in culture medium for 40 minutes at room temperature. Subsequently, the medium was removed, and compound 11 was added in PBS titrated to an acidic pH with citric acid. After 2 minutes at room temperature, the acidic medium was removed, the cells were washed three times with PBS, and incubated for 4 hours at 37°C before fixation with 4% PFA. Nuclei were stained with 4′,6-diamidino-2-phenylindole (DAPI; Molecular Probes) and fluorescence microscopy images were captured using a Nikon Eclipse 80i Fluorescence microscope equipped with a DS-Qi1Mc camera with a 100× magnification.

Image analysis was followed to quantify syncytium formation in each treatment. Briefly, nuclei and GFP-expressing cells were segmented in every image using local threshold and watershed algorithms. Afterward, GFP-expressing cells were manually classified into syncytium or non-fused cells based on the number of nuclei and morphology. All cells with one nucleus were automatically considered an individual cell. For every image, a fusion index was calculated as 1 − *C*/*N*, where *C* is the number of GFP-expressing cells and *N* is the number of nuclei within GFP-expressing cells in the image. Six random fields were taken per treatment and three independent experiments were performed.

### Plaque assay

Confluent Vero cells in 24-well plates were infected with serial dilutions of viral samples and were incubated for 1 h at 37°C. Afterward, 1 mL of overlay (DMEM, 2% FBS, 0.4% methylcellulose) was added to each well. Three days post-infection, cells were fixed with 10% formaldehyde and were stained with crystal violet solution (20% ethanol, 0.1% crystal violet in water) to allow plate lysis count.

### Growth curves

Confluent Vero or C6/36 cells grown in 24-well plates were infected with WT or mutant CHIKV-ZsGreen at an MOI of 0.01. After incubating for 1 hour at 37°C for mammalian cells infection or 28°C for mosquito cells infection, the inoculum was removed, and the cells were washed three times with PBS. 750 µL of culture medium was added, and the cells were incubated for 3 days at the corresponding growth temperature (see “Cells and Viruses”). At various time points, a 50 µL aliquot was removed, replaced with culture medium, and stored at −70°C. The viral titer at each time point was determined by plaque assay.

### Viral yield inhibition assay

Confluent Vero cells were treated with increasing concentrations of compound 11 and were infected with CHIKV-ZsGreen WT or mutant viruses at an MOI of 0.01. Cells were incubated for 1 hour at 37°C and then overlaid with culture medium containing the corresponding concentration of compound 11. Two days after the infection, the number of viral particles in the supernatant was titrated by plaque assay.

### Competition assay

Virus stocks were diluted and mixed at a 1:1 ratio to infect confluent Vero cells grown in 24-well plates at an MOI of 0.01. Supernatants of cells treated with compound 11 at 50 µM or mock-treated were collected 3 days after the infection, and RNA was extracted using the Quick RNA Viral kit (Zymo Research). The cDNA corresponding to the regions coding for E2 and E1 was obtained by RT-PCR using SuperScript III reverse transcriptase (Invitrogen) and primers 137 (5′-CGTTTGTAGATAACTGCGG-3′) and 95 (5′-TACTTAATTGTCGAGCTCTTAGTGCCTGCTGA-3′) for first-strand synthesis of E2 and E1, respectively. Then, Pfx Accuprime polymerase (Invitrogen) and primers 136 (5′-GAAGAGTGGAGTCTTGCC-3′) and 137, and 93 (5′-GACTGAAGGGCTCGAGGTCA-3′) and 95 were used for PCR amplification of E2 and E1 fragments, respectively. PCR amplicons were sequenced by the Sanger method and chromatograms analyzed using 4Peaks software (Nucleobytes).

### Molecular dynamics and residue interaction network

We performed molecular dynamics simulations of the CHIKV pre-fusion E1-E2 heterodimer in solution (PDB 3N42) using GROMACS 2021.2 (38). The protonation state at pH 7 of all protonable residues was defined using PROPKA (39). The Amber99SB*-ILDN force field (40, 41) was used to describe the protein, and a cutoff value of 10 Å was used for short-range electrostatic and van der Waals interactions. Long-range electrostatic interactions were treated with PME (Particle Mesh Ewald). The system was solvated using a dodecahedral box of TIP3P water (https://conan.io/) extending 12 Å from the protein’s surface. The system was neutralized with 0.15 M NaCl. After, the system was minimized, heated to 310 K, and equilibrated for 200 ps in the NVT ensemble using the V-rescale thermostat (39) with a coupling constant of 0.1 ps. A second equilibration step of 1 ns was carried out in the NPT ensemble using the Berendsen barostat (https://networkx.org/) with a reference pressure of 1 bar and a coupling constant of 2 ps. Finally, the production run was conducted in the NPT ensemble using the V-rescale thermostat and the Parrinello-Rahman barostat (42). In the equilibration and production runs, the LINCS algorithm (43) was used, and a time step of 2 fs was employed. Four independent runs of 250 ns were carried out with the WT protein. For analysis, we concatenated the last 200 ns of each run. The molecular dynamics simulations were carried out on high-performance computing centers CCAD (https://ccad.unc.edu.ar/) and the High-Performance Computing Portal at the NYU Grossman School of Medicine.

From the concatenated trajectory, a PCA was conducted with GROMACS tools. The RIN was built from the contact matrix and the DCCM following the guidelines outlined in the work of Sethi and colleagues (23). The contact matrix was obtained using the CONAN tool (https://conan.io/). Two residues were considered to be in contact if they remained within 4.5 Å of each other for more than 75% of the dynamics. The DCCM was derived from the covariance matrix obtained in the PCA with GROMACS. The RIN was constructed using a Python script based on the NetworkX package (https://networkx.org/). A node was defined for each residue, and an edge was established between two nodes if they were in contact during the dynamics. The weight of each edge was defined as the information transfer probability *d_ij_* obtained from the correlation of each pair of residues in the dynamics (22). From the RIN, the betweenness centrality of each edge was calculated, and the residue communities were identified using the Girvan-Newman algorithm (24) with NetworkX methods.

### Viral particle stability

WT or mutant CHIKV-ZsGreen stocks were diluted to 5 × 10^4^ PFU/mL in serum-free DMEM. At different time points (0, 8, and 24 hours), an aliquot was frozen at −70°C, and the viral titer for each treatment was quantified by plaque assay.

### Viral attachment

Confluent BHK or Vero cells grown in 12-well plates were infected with WT or mutant CHIKV-ZsGreen at an MOI of 0.1. After incubating for 40 minutes at room temperature, cells were washed three times with PBS and harvested in culture medium using a cell scraper. Cells were lysed by three cycles of freezing in liquid nitrogen and thawing at 37°C, and the supernatant was clarified by centrifugation for 10 minutes at 1,000 × *g* and 4°C. The number of viral particles in the clarified supernatant was determined by plaque assay.

### Cholesterol dependence

A 25 mM MβCD (Thermo Fisher) solution in α-MEM with 25 mM HEPES at pH 7.5 was prepared for immediate use. Confluent BHK cells were treated with increasing concentrations of MβCD and were incubated for 1 hour at 37°C. Subsequently, cells were washed three times with PBS and were infected with CHIKV-ZsGreen WT or mutant viruses at an MOI of 0.1. After incubating for 1 hour at 37°C, the inoculum was removed, the cells were washed three times with PBS and medium containing 20 mM NH_4_Cl was added to prevent reinfection. One day post-infection, the percentage of infected cells was determined by flow cytometry.

### Experimental infection in mice

Four- to five-week-old male and female C57BL/6J mice (*n* = 10 per group) were infected subcutaneously in the footpad with 1,000 PFU of WT or each of the mutant CHIKV-ZsGreen. At 2 days post-infection, mice were euthanized to collect blood via cardiac puncture and to harvest the footpad. The footpad was ground in 1 mL of DMEM containing 2% FBS with steel beads using a Tissue-Lyser II (Qiagen), and debris was clarified by centrifugation at 8,000 × *g* for 10 minutes. Viral titers in the footpad were quantified by plaque assay on Vero cells. For serum, whole blood was centrifuged at 4,000 × *g* for 15 minutes, and the serum was placed in Trizol. RNA was extracted following the manufacturer’s instructions, and CHIKV genomes were quantified by RT-qPCR (Applied Biosystems RNA-to-Ct one-step kit) with the following primers targeting CHIKV nsP4: 5′-TCACTCCCTGCTGGACTTGATAGA-3′ and 5′-TTGACGAACAGAGTTAGGAACATACC-3′, and probe: 5′-(6-carboxyfluorescein)-AGGTACGCGCTTCAAGTTCGGCG-(black-hole quencher)-3′. *In vitro* transcribed CHIKV RNA was used to generate a standard curve. All RT-qPCR samples were run in technical duplicates. All mouse experiments were performed in the biosafety level 3 facility ABSL3 at the NYU Grossman School of Medicine.

### Experimental infection in mosquitoes

Four to seven–7 days post-emergence, female *Ae. aegypti* mosquitoes (Poza Rica, Mexico; F40) were fed a blood meal containing 10^6^ PFU/mL of WT or each of the mutant CHIKV-ZsGreen supplemented with 5 mM ATP for 30 minutes. Engorged females were sorted and incubated at 28°C, 70% humidity, and 12 h diurnal light cycle with 10% sucrose *ad libitum* for 7 days. After incubation, whole mosquitoes were ground in 300 mL of PBS with ceramic beads using a Tissue-Lyser II (Qiagen), and debris was removed by centrifugation at 8,000 × *g* for 10 minutes. Viral titers in the bodies were quantified in the bodies by plaque assay. All mosquito studies were performed in the NYU Grossman School of Medicine ABSL3 facility.
